# The Structural Basis for mRNA Recognition and Cleavage by the Ribosome-Dependent Endonuclease RelE

**DOI:** 10.1016/j.cell.2009.11.015

**Published:** 2009-12-11

**Authors:** Cajetan Neubauer, Yong-Gui Gao, Kasper R. Andersen, Christine M. Dunham, Ann C. Kelley, Jendrik Hentschel, Kenn Gerdes, V. Ramakrishnan, Ditlev E. Brodersen

**Affiliations:** 1 MRC Laboratory of Molecular Biology, Cambridge CB2 0QH, UK; 2 Department of Molecular Biology, Aarhus University, DK-8000 Aarhus C, Denmark; 3 Institute for Cell and Molecular Biosciences, The Medical School, University of Newcastle, Newcastle NE2 4HH, UK

**Keywords:** PROTEINS

## Abstract

Translational control is widely used to adjust gene expression levels. During the stringent response in bacteria, mRNA is degraded on the ribosome by the ribosome-dependent endonuclease, RelE. The molecular basis for recognition of the ribosome and mRNA by RelE and the mechanism of cleavage are unknown. Here, we present crystal structures of *E. coli* RelE in isolation (2.5 Å) and bound to programmed *Thermus thermophilus* 70S ribosomes before (3.3 Å) and after (3.6 Å) cleavage. RelE occupies the A site and causes cleavage of mRNA after the second nucleotide of the codon by reorienting and activating the mRNA for 2′-OH-induced hydrolysis. Stacking of A site codon bases with conserved residues in RelE and 16S rRNA explains the requirement for the ribosome in catalysis and the subtle sequence specificity of the reaction. These structures provide detailed insight into the translational regulation on the bacterial ribosome by mRNA cleavage.

## Introduction

Rapid adaptation to environmental stress is vital for free-living bacteria. During deprivation of nutrients, uncharged transfer RNAs (tRNAs) bind to the ribosome and activate the stringent factor, RelA, which recognizes stalled ribosomes and catalyzes synthesis of the signal nucleotide, (p)ppGpp. This alarmone regulates the *stringent response*, a far-reaching adaptation of the bacterial metabolism to the changing growth conditions (Potrykus and Cashel, 2008). The stringent response also leads to activation of RelE, an effective inhibitor of protein synthesis. Under normal physiological conditions, RelE forms a tight complex with another protein, RelB, and is inactive (Christensen and Gerdes, 2003; Christensen et al., 2001; Galvani et al., 2001; Gotfredsen and Gerdes, 1998; Li et al., 2009; Overgaard et al., 2008; Pedersen et al., 2003). After the controlled degradation of RelB by Lon protease, RelE is able to bind the ribosome and specifically cleave messenger RNA (mRNA) in the A site (Christensen and Gerdes, 2003; Christensen et al., 2001; Galvani et al., 2001; Li et al., 2009; Pedersen et al., 2003).

Such toxin-antitoxin pairs are very common in bacteria, and the RelE superfamily also encompasses HigB, YoeB, YafQ, and YhaV, associated with a variety of cellular response mechanisms (Christensen-Dalsgaard and Gerdes, 2006; Grady and Hayes, 2003; Prysak et al., 2009; Schmidt et al., 2007). The crystal structure of YoeB showed that its fold and catalytic mechanism resembles compact bacterial endonucleases like RNase T1 (Kamada and Hanaoka, 2005). RelE is structurally similar to YoeB (Li et al., 2009; Takagi et al., 2005) but lacks the conserved catalytic histidine and glutamate residues promoting the idea that it might work by activating an intrinsic nuclease activity of the ribosome (Garza-Sanchez et al., 2008; Hayes and Sauer, 2003; Kamada and Hanaoka, 2005; Li et al., 2009; Sunohara et al., 2004; Takagi et al., 2005). Although this could explain how pausing ribosomes are recovered for rescue by tmRNA in the absence of stringent response factors (Hayes and Sauer, 2003; Kamada and Hanaoka, 2005; Sunohara et al., 2004), recent data suggest that cleavage is most likely a result of the combined action of RelE-like endonucleases and exonucleases like RNase II (Garza-Sanchez et al., 2009). RelE-induced cleavage most commonly occurs after the second nucleotide of the A site codon, although it is occasionally also seen after the third nucleotide and, upon peptide release, even in the E site (Pedersen et al., 2003). The mRNA sequence appears to have a significant effect on the cleavage efficiency, with the UAG and UGA stop codons and sense codons like UCG and CAG, among the most efficiently cleaved (Pedersen et al., 2003). Together, the data suggest a subtle preference for pyrimidines in the first position of the codon, and purines in positions two and three (Pedersen et al., 2003).

To investigate the mechanism of ribosome-dependent mRNA recognition and cleavage by RelE, we have determined the isolated crystal structure of *Escherichia coli* RelE at 2.5 Å resolution and structures of RelE bound to *Thermus thermophilus* 70S ribosomes in complex with mRNA and tRNA^fMet^ in the pre- and postcleavage states at 3.3 and 3.6 Å resolution, respectively. The structures reveal that RelE only undergoes slight conformational changes upon binding the ribosome, where it occupies the A site on the 30S subunit. Binding of RelE significantly reorganizes the mRNA of the A site, leading to 2′-OH-induced hydrolysis between codon positions two and three. The structure suggests a model in which RelE uses conserved basic residues to promote activation of the RNA 2′-OH, stabilize of the transition state, and protonate of the 5′ leaving group of the reaction, and provides a firm basis for understanding both the cleavage mechanism and sequence specificity of RelE and other related ribosome-dependent endonucleases.

## Results and Discussion

### Structure Determination

Wild-type RelE (RelE^wt^) and an inactive mutant, RelE^R45A/R81A^, were expressed in complex with RelB and isolated by denaturation followed by renaturation (Pedersen et al., 2002). Intact *T. thermophilus* 70S ribosomes were purified as previously described (Selmer et al., 2006) and incubated with RelE, *E. coli* tRNA^fMet^, and mRNA, either unmodified or 2′-O-methylated at all three nucleotides of the A site codon, prior to crystallization. Under these conditions, RelE^wt^ cleaves the unmodified mRNA inside the ribosome, and complexes representing the uncleaved (2′-O-methyl mRNA + RelE^R45A/R81A^) and cleaved (unmodified mRNA + RelE^wt^) states were subsequently crystallized in space group P2_1_2_1_2_1_ with two ribosomes per asymmetric unit (Selmer et al., 2006). In this crystal form, the ribosomes contain a noncognate tRNA^fMet^ bound to the AAA codon in the E site, tRNA^fMet^ bound to the AUG codon in the P site, and UAG in the A site, a stop codon which has previously been shown to elicit efficient cleavage by RelE on the *E. coli* ribosome in vitro (Pedersen et al., 2003).

**Figure 1:**
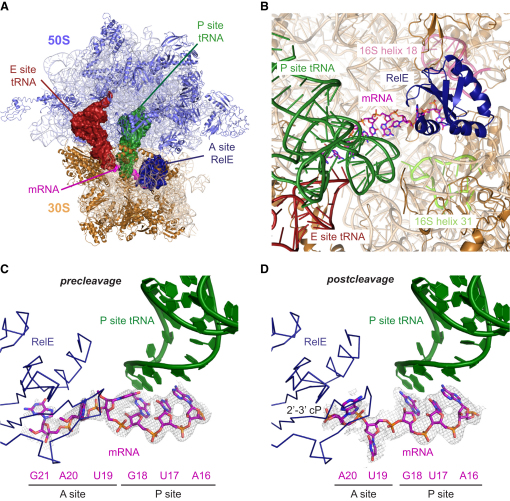
Overview of the RelE-Bound 70S Ribosome (A) Top view of the 70S ribosome with the 50S (blue) and 30S (wheat) subunits surrounding RelE (A site, blue), tRNA^fMet^ (P site, green), a noncognate tRNA^fMet^ (E site, red), and mRNA (magenta). (A)–(C) are based on the precleavage structure. (B) Close-up of the A and P sites of the 30S subunit viewed from the interface to the 50S. RelE (blue cartoon) spans the 16S rRNA from the head (helix 31 region, green) to the body (helix 18, pink). The mRNA is shown in purple sticks, and the P and E site tRNAs colored as in (A). (C) Close-up view of the A and P sites showing RelE (blue Cα trace), mRNA (purple sticks), and P site tRNA (green cartoon) along with the DF_o_-mF_c_ electron density of the precleavage structure contoured at 1.5 σ. The mRNA sequence is indicated. (D) The postcleavage structure showing the position of the 2′-3′ cyclic phosphate generated upon cleavage (2′-3′ cP). The map is contoured at 1.2 σ. See also Figure S1.

Difference electron density maps calculated after initial refinement of diffraction data extending to 3.3 Å (2′-O-methyl mRNA + RelE^R45A/R81A^) and 3.6 Å (unmodified mRNA + RelE^wt^) using a model containing only ribosomal RNA (rRNA) and ribosomal proteins allowed modeling of mRNA and RelE in the two states (Table 1, and Figure S1 available online). The final RelE models are complete for residues 2–95 (Figures 1A and 1B). All three nucleotides of the A site codon (UAG) and two downstream residues (AA) could be traced in the structure determined with 2′-O-methyl mRNA and RelE^R45A/R81A^, representing a precleavage state (Figure 1C). In the structure determined using RelE^wt^ and unmodified mRNA, cleavage has occurred prior to crystallization, and there is weak but consistent density for just positions 1 and 2 of the A site codon, thus representing a postcleavage state (Figure 1D). Final refinement of the structures including mRNA and RelE gave crystallographic R/R_free_ = 21.9% / 24.7% (precleavage) and R/R_free_ = 21.5% / 24.8% (postcleavage).

To analyze possible conformational changes in RelE upon binding the ribosome, we also determined the crystal structure of *E. coli* RelE in its isolated form. Untagged RelE^R81A^ was expressed in complex with His-RelB, separated from His-RelB, and purified to homogeneity. The untagged form of RelE crystallized in the monoclinic space group P12_1_1 and diffraction data were collected to 2.5 Å. The structure was determined by molecular replacement using the nuclear magnetic resonance (NMR) structure of RelE (Li et al., 2009) and has three molecules in the crystallographic asymmetric unit. Refinement was performed using individual B factors with final crystallographic R/R_free_ = 22.6% / 26.8%.

### Overall Structures of Isolated and Ribosome-Bound RelE

*E. coli* RelE is an 11.2 kDa protein consisting of a compact, four-stranded antiparallel β sheet flanked by three α helices (Figure S1B). The crystal structure of isolated RelE is very similar to the structure determined by NMR (Li et al., 2009), but minor differences are observed in the loops and the C-terminal helix, which is absent in archaeal homologs of RelE and appears to be the most flexible part of the structure (Figures S1 and S3) (Takagi et al., 2005). Upon binding the ribosome, RelE maintains its compact conformation, although slight rearrangements are seen where interactions are made to rRNA and mRNA (Figure S1B). Consequently, the conformation of RelE that arises immediately after degradation of RelB represents the active form of the toxin.

RelE occupies the A site in near identical conformations in both the pre- and postcleavage structures and blocks access of translation factors and tRNA to the ribosome (Figure 1B). With extended, basic residues on both sides of its compact domain, RelE precisely spans the distance and complements the shape of the space between the head and the body of the 30S subunit and makes direct contacts to helices 30–31 and helix 18 (the 530 region) of 16S ribosomal RNA (rRNA). Superposition of the structure of A site-bound tRNA shows that the side of RelE facing the P site tRNA very closely mimics the shape of A site tRNA, while the opposite side is wider and closely complements the structure of the 16S rRNA.

### RelE Interacts Tightly with Several Domains of 16S Ribosomal RNA

**Figure 2:**
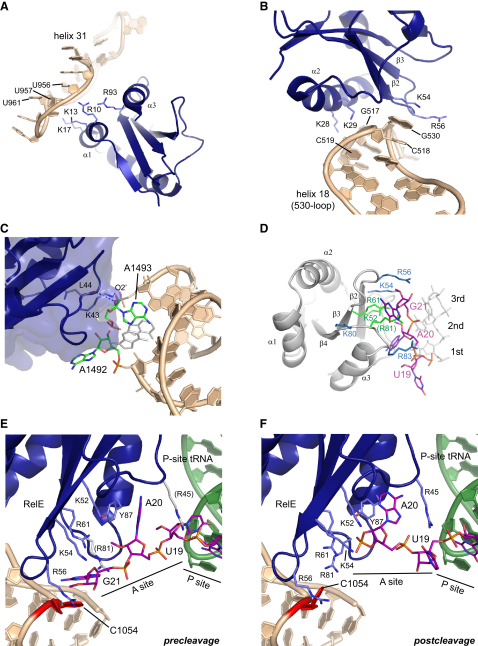
Interactions with rRNA and mRNA (A) Interactions between RelE (blue) and the head region of the 30S (helix 31, wheat). (A)–(E) are based on the precleavage structure. All rRNA references correspond to the *E. coli* sequences. (B) Interactions between RelE and the body region of the 30S (helix 18, wheat). (C) Interactions between RelE (blue, semitransparent surface with interacting residues shown as sticks) and the decoding center bases A1492/A1493 (green sticks) of 16S rRNA helix 44 (wheat). The unbound conformation of the decoding site is shown with white sticks. (D) Overview of the contacts between RelE and mRNA. RelE is shown as a white cartoon with basic residues near the mRNA as blue sticks. Residues known to affect the activity of *P. horikoshii* RelE are shown in green. The normal mRNA path is shown with white sticks and the RelE-bound mRNA path with purple sticks. The position of the R81 side chain is inferred from the Cβ position. (E and F) Details of the interactions with mRNA in the precleavage (E) or post-cleavage (F) state showing RelE (blue) with relevant side chains as sticks, P site tRNA (green cartoon), the 16S conserved rRNA helix 34 bulge (wheat), and the mRNA (purple sticks) with the A and P site codons labeled. C1054 is shown in red. The positions of the side chains of R45 and R81 are inferred from Cβ positions and are therefore shown in gray. See also Figure S2.

**Figure 3:**
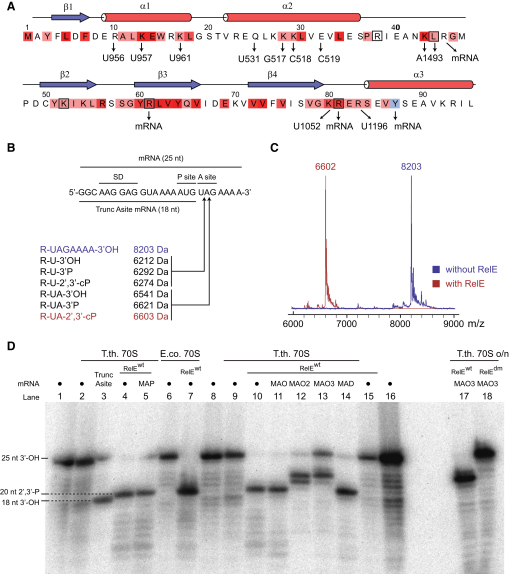
In Vitro mRNA Cleavage Assay on the 70S Ribosome (A) Sequence of *E. coli* RelE with the conservation among homologs indicated as increasing strength of red color and the conserved tyrosine at the C terminus in light blue. The secondary structure is shown above the sequence and the interactions to rRNA and mRNA below (all numbers correspond to the *E. coli* 16S sequence). Residues in the *P. horikoshii* RelE homolog that affect the activity are indicated with black boxes (Takagi et al., 2005). (B) Overview of the mRNAs used for the in vitro cleavage assays. The 25 nt mRNA consists of a Shine-Dalgarno element (SD) followed by a spacer and the P site (AUG) and A site (UAG) codons. “Trunc Asite” ends after the P site codon with a 3′-OH. The table shows predicted masses of full length mRNA and fragments that would result from cleavage after position 1 or 2 of the A site codon leaving either a 3′-OH, 3′-phosphate (3′-P), or 2′-3′ cyclic phosphate (2′,3′-cP). (C) MALDI mass spectrometry spectra and masses of RNA fragments isolated from complexes in the absence (blue) or presence (red) of RelE. (D) In vitro cleavage assay using 5′ ^32^P-labeled mRNA substrates. • is the 25 nt unmodified mRNA; MAP has phosphorothioate linkages after A site codon positions 1 and 2; MAO, MAO2, and MAO3 are 2′-O-methylated at position 1, positions 1 + 2, or all three positions, respectively; and MAD contains a deoxyribose at position 1. The mRNAs were incubated with either *T. thermophilus* (T.th.) or *E. coli* (E.co.) 70S ribosomes, tRNA^fMet^, and either RelE^wt^ or RelE^R81A/R45A^ (RelE^dm^) as indicated for either 1 hr (lanes 1–16) or overnight (lanes 17 and 18). The size markers indicate the positions of the full-length (25 nt 3′-OH) and Trunc Asite (18 nt 3′-OH) RNAs and the 20 nt 2′-3′ cyclic phosphate cleavage product, which runs approximately 1 nt faster than the corresponding 3′-OH species. See also Figure S3.

*E. coli* RelE contains 21 basic residues (out of 95) and thus possesses a large potential for interacting with negatively charged rRNA (Figures 2 and 3A). In both the pre- and postcleavage structures, we observe similar, tight interactions between RelE and 16S rRNA, while contacts to the 50S subunit and ribosomal proteins are almost absent (Figure 2). A large number of basic residues located on the three helices interact with 16S rRNA. Of these, residues located in α1 and α3 mainly contact the helix 31 region of the head of the 30S subunit (Figure 2A), while those on α2 point in the opposite direction and interact with the 530 region, 16S helix 18 (Figure 2B). Important interactions involving residues in α1 include K13 that interacts with the O4 of U957 and K17 that contacts the phosphate of U961, all in the helix 31 region (all references to rRNA in this paper correspond to the *E. coli* sequences; Figures 2A and 3A). Interactions between residues in α2 and the body of the 30S include K28 and K29, which contact the 2′-OH and phosphate oxygen atoms of residues G517 and C518 of the 530 loop, respectively (Figure 2B).

RelE also interacts tightly with the 30S decoding center via the loop between α2 and β2, causing a significant change in the conformation of the conserved adenosines, A1492 and A1493, known to be directly involved in the decoding of mRNA during translation (Figure 2C) (Ogle et al., 2001). Using its side chain amino group, K43 contacts the 2′-OH of A1493, while the main chain carbonyl of L44 interacts with a phosphate oxygen atom (Figure 2C). Interestingly, this KL motif is conserved in many RelE homologs, and mutation of L44 in the archaeal RelE homolog has been shown to significantly reduce activity (Figures 3A and S3) (Takagi et al., 2005). As a result of RelE binding, A1492 is pulled fully out of 16S helix 44, while A1493 remains partly extruded and stacks on 23S nucleotide A1913 in the only contact to the 23S rRNA (Figure 2C). The conformation of A1492/A1493 is distinctly different from that observed in empty ribosomes or when cognate tRNA is bound in the A site (Ogle et al., 2001; Selmer et al., 2006; Wimberly et al., 2000) but reminiscent of the conformation seen in the presence of release factors (Figure S2) (Laurberg et al., 2008; Weixlbaumer et al., 2008). The close interaction between RelE and several highly conserved regions of 16S rRNA is consistent with the observation that RelE homologs from archaea are functional in *E. coli* (Christensen and Gerdes, 2003) and, conversely, that *E. coli* RelE also is capable of cleaving mRNA on the eukaryotic ribosome (Andreev et al., 2008).

### RelE Induces a Change in the Conformation of mRNA that Promotes Cleavage

Recognition of mRNA involves several highly conserved residues on the β sheet of RelE located in a cleft formed between the β sheet and α3. The region is packed with basic side chains (Figure 2D, blue), and the most central of these have been shown to be of critical importance for the activity of *P. horikoshii* RelE (Figure 2D, green) (Takagi et al., 2005). The overall path of the mRNA through the RelE-bound ribosome deviates significantly from that observed in the tRNA-bound state of the ribosome with shifts, as measured by the distances between identical C1′ atoms of 7.0, 7.6, and 8.3 Å for the three A site nucleotides, respectively (Figure 2D) (Selmer et al., 2006). In the precleavage structure, two nucleotides downstream of the A-site codon are visible in the electron density, and their position is consistent with the general path of mRNA through the 30S subunit (Yusupova et al., 2006). However, the mRNA path inside the RelE-bound ribosome is longer than usual and likely requires one or more additional nucleotides to be threaded through the entry channel in the 30S subunit.

When the A site codon interacts with RelE, the bases in positions 2 and 3 are splayed apart as conserved basic side chains interact with the phosphate group between them. This rearrangement is critical as it helps to orient the mRNA optimally for catalysis. The reorientation of the nucleotides is stabilized by stacking of the base at position 2 (A20) with a conserved tyrosine (RelE Y87) and the base at position 3 (G21) with C1054 of 16S rRNA (Figures 2E and S1C). C1054 is part of a highly conserved bulge in 16S helix 34, and mutation of this nucleotide seriously affects ribosomal function by causing read-through of nonsense codons (Murgola et al., 1988; Prescott et al., 1991). In the postcleavage structure, A20 maintains the stacking interaction with Y87 but has moved slightly back toward the normal mRNA path (Figure 2F).

### RelE Cleaves the mRNA after the Second Position of the A Site Codon

Next, we decided to confirm that cleavage occurs at the observed position under the conditions used for crystallization by analyzing RelE-mediated cleavage of the mRNA on the ribosome in vitro. *E. coli* or *T. thermophilus* 70S ribosomes were assembled in the same way as for structure determination, but with 5′-^32^P-labeled mRNAs. After incubation in the presence of RelE, resulting RNA fragments were isolated by gel purification and analyzed by matrix-assisted laser desorption ionization (MALDI) mass spectrometry. Spectra collected from the samples incubated in the absence of RelE showed of a peak with mass 8203 Da corresponding to the full-length mRNA (Figure 3C, blue), while a mass of 6602 Da was observed for samples containing RelE, confirming that cleavage occurs following the second position of the A site codon (Figures 3B and 3C, red).

We also analyzed the resulting mRNA fragments by denaturing gel electrophoresis by comparison to a reference RNA completely synthesized without A site codon (“Trunc Asite mRNA”) (Figure 3D). Incubation of programmed *E. coli* or *T. thermophilus* ribosomes with RelE^wt^ consistently resulted in RNA fragments appearing 1 nucleotide longer than the reference RNA (Figure 3D, lanes 3, 4, and 7), while incubation of the mRNA alone or in the presence of either programmed 70S ribosomes or RelE^wt^, but not both, did not (Figure 3D, lanes 1, 2, 6, and 15). Although this could be interpreted as cleavage after position 1 of the A site codon, it must be borne in mind that RNAs arising from hydrolysis by internal attack of the 2′-OH on the phosphate contain an additional 3′ phosphate group compared to synthesized RNAs and typically run between ½ and 2 nucleotides faster than their 3′-OH counterparts (Cruz-Reyes et al., 1998). The combined results are therefore consistent with a model whereby cleavage occurs at position 2 of the A site codon by a mechanism involving the 2′-OH as an internal nucleophile.

### mRNA Cleavage by RelE Follows a Mechanism Involving 2′-OH-Induced Hydrolysis

To precisely define the involvement of the ribose 2′-OH of mRNA in the cleavage reaction, we repeated the experiments using RNAs modified by 2′-O-methylation at A site codon position 1 (MAO), positions 1 and 2 (MAO2), or all three positions (MAO3), containing a 2′-deoxy ribose at position 1 (MAD), or phosphorothioate linkages after positions 1 and 2 (MAP) (Figure 3D). RelE cleaved both the phosphorothioate-modified RNA (lane 5) as well as mRNAs containing a 2′-O-methyl group (MAO, lane 11) or a 2′-deoxyribose at position 1 of the A site codon (MAD, lane 14) to the same extent as unmodified RNA, showing that the 2′-OH of the first nucleotide of the codon is not directly involved in the reaction. In contrast, 2′-O-methylation of both positions 1 and 2 (MAO2, lane 12) or all three codon positions (MAO3, lane 13) shifted the cleavage position downstream to the first or second unmodified nucleotide, indicating that RelE only cleaves at positions where the 2′-OH is available. Surprisingly, the mutant RelE^R45A/R81A^ did cleave mRNA to some extent (data not shown), but not the mRNAs modified by 2′-O-methylation at all three positions even after overnight incubation (lane 18), suggesting that the same conserved basic residues are involved in all the cleavage events observed. This strongly suggests that there is only a single active site in RelE and, consequently, that the mRNA most likely shifts inside the ribosome to allow cleavage in other places than between A site position 2 and 3. Ultimately, it is possible that even cleavage of mRNA in the ribosomal E site is a result of such mRNA frame-shifting (Pedersen et al., 2002). On the other hand, our results also demonstrate that R81, which is one of the most conserved amino acids in RelE and is known to be required for efficient inhibition of translation in vivo (Pedersen et al., 2002), is not absolutely essential for catalysis, but greatly enhances the reaction rate.

The involvement of the 2′-OH in the reaction can be confirmed further by close inspection of the mass spectrometry spectra (Figure 3C). After cleavage, we observe an RNA fragment with a mass of 6602 Da, more closely matching a situation where cleavage occurs after the second position of the A site codon with a 3′-phosphate (6621 Da) than a 3′-OH (6541 Da, Figure 3B). But most precisely, the measured mass matches that of a cyclic 2′-3′ phosphate (predicted to 6603 Da), which lacks an oxygen atom compared to a 3′-phosphate monoester and can only be generated by internal attack of the 2′-OH. The combined results therefore strongly support a model in which RelE-mediated cleavage of mRNA occurs after position 2 of the A site codon by a 2′-OH-induced hydrolytic mechanism, leading to a 2′-3′ cyclic phosphate at the newly formed 3′ end and a 5′-OH. In addition, and unlike other structurally related nucleases such as RNase T1, RelE does not appear to catalyze the subsequent hydrolysis of the cyclic phosphate to 3′-phosphate (Heinemann and Saenger, 1983).

### mRNA Cleavage Requires Both RelE and the Ribosome

RelE is structurally related to a group of sequence-specific, extracellular microbial endoribonucleases that cleave polyribonucleotides independently of the ribosome specifically after guanosine residues, but it lacks their conserved active site residues (Heinemann and Saenger, 1983; Li et al., 2009; Takagi et al., 2005). Structural alignment of RNase T1 bound to two guanosine molecules representing a postcleavage state (Zegers et al., 1994) with RelE bound to mRNA, however, reveals a similar position and to some extent orientation of nucleotides 2 and 3 of the A site codon in the precleavage structure to the guanosine residues in RNase T1 (Figure S4A). This observation suggests that RelE nevertheless shares the location of its active site with the microbial RNases and, consequently, that it might recognize RNA by a similar mechanism.

**Figure 4:**
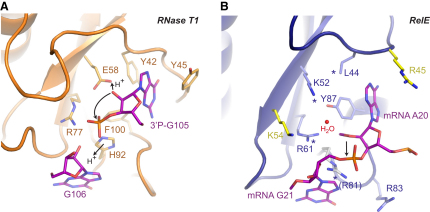
Comparison of the Active Sites of RelE and RNase T1 (A) The active site of RNase T1 (orange cartoon) with critical amino acids shown as labeled sticks. The 3′-phosphate guanosine representing the 3′ end of the cleaved RNA and the guanosine representing the 5′ end are shown as purple sticks. Movements of protons during the reaction are shown with straight arrows (marked H^+^), and the nucleophilic attack of the 2′ oxyanion with a curved arrow. (B) The corresponding region in RelE (blue cartoon) with residues implied in catalysis as blue sticks, and other nearby basic residues as yellow sticks. Residues critical for the activity of *P. horikoshii* RelE are marked with a ^∗^ (Takagi et al., 2005). The nucleotides at positions 2 and 3 of the A site codon are shown as purple sticks and the scissile bond is marked with an arrow. The water molecule near the 2′-OH of position 2 (A20) is shown as a red sphere. See also Figure S4.

The reaction mechanism proposed for RNase T1 involves E58 acting as a general base to activate the 2′-OH of guanosine for an S_N_2-type nucleophilic attack on its 3′ phosphate group, H92 (general acid) stabilizing the leaving 5′-OH, and formation of a 2′-3′ cyclic phosphodiester intermediate product (Figure 4A) (Heinemann and Saenger, 1983). In RNase T1, correct orientation of the substrate for inline attack is achieved by “sandwiching” of the guanine base between Y42 and Y45, similar to the stacking of Y87 of RelE with the second base of the A site codon (Figure 4). It can be noted that Y87 is shifted about 14 Å away in the RelE-RelB complex, thus effectively destroying the active site when the antitoxin is bound. In addition, Y87 is the closest functional group to the 2′-OH of the second A site nucleotide in the ribosome-bound structure (3.8 Å), suggesting that it could be important for the catalytic function of RelE. In RNase T1, the downstream nucleotide is held tightly in place by an aromatic stack formed by H92 and F100, but such aromatic residues are absent in RelE and the nucleotide is instead stabilized by stacking with C1054 of 16S rRNA. The structurally related YoeB, which has retained both the active site histidine (H83) and glutamate (E46) and therefore most probably employs the classical cleavage mechanism, has been shown to be able to cleave RNA independently of the ribosome in vitro (Kamada and Hanaoka, 2005). However, recent evidence suggests that it only acts on the ribosome in vivo (Christensen-Dalsgaard and Gerdes, 2008), and comparison with RelE reveals that YeoB also lacks the hydrophobic stack required for orientation of the nucleotide downstream of the cleavage point. It may therefore be that it is the combination of a noncanonical active site and lack of the hydrophobic stack that together leads to the strict requirement for the ribosome in the case of RelE. In summary, structural comparison suggests that RelE cleaves mRNA with an active site related to that used by extracellular bacterial endonucleases like RNase T1 and, consequently, that the ribosome is not directly involved in catalysis. However, the involvement of 16S rRNA in orienting the mRNA correctly for the cleavage reaction rationalizes why the ribosome is critical for RelE activity.

### A Mechanism for the Ribosome-Dependent mRNA Cleavage by RelE

RelE contains no functional equivalents of H92 and E58 that are required for catalysis in RNase T1, but instead depends on a set of conserved basic residues (e.g., K52, R61, and R81) for activity, as shown for the archaeal RelE (Figure S3) (Takagi et al., 2005). In ribosome-bound RelE, most of these residues (Figure 4B, marked with ^∗^) cluster around the scissile phosphate ester bond (Figure 4B, arrow) and the stack between Y87 and the second base of the A site codon, but their involvement in catalysis has so far been unclear. However, cleavage of RNA by attack of the 2′-OH is a chemically well understood mechanism employed by many ribozymes, and a set of general requirements can be defined (Cochrane and Strobel, 2008). First, the 2′-OH must be activated by abstraction of the proton by a general base, allowing it to act as a nucleophile and the RNA to orient itself for an inline attack at the phosphate; second, the negatively charged, trigonal bipyramidal transition state must be stabilized; and third, the leaving 5′-OH group should be stabilized through donation of a proton by a general acid. Superposition of RelE with RNase Sa2, another related bacterial endonuclease, reveals that the general base in RNase Sa2, E56 (E58 in RNase T1), aligns with K52 in RelE, while the general acid, H86 (H92 in RNase T1), coincides with R81 (Bauerova-Hlinkova et al., 2009) (Figure S4B). In addition, R61 and K54 in RelE align well with two arginine residues critical for stabilizing the transition state in RNase Sa2, R71 and R67, and are both conserved in many RelE homologs, suggesting that these residues may play a similar role here.

**Figure 5:**
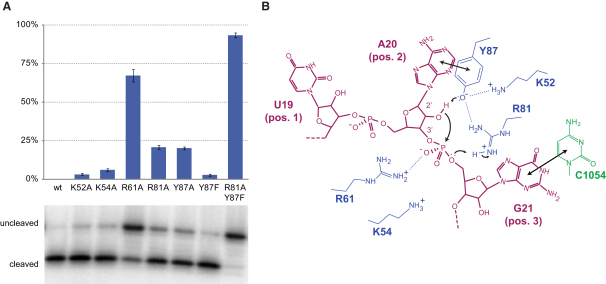
A Mechanism for Ribosome-Dependent mRNA Cleavage by RelE (A) Relative inhibition of cleavage in RelE mutants shown as % uncleaved mRNA after 15 min incubation at 37°C, mean value ± SEM. Below, a denaturing RNA gel showing substrate and products for each mutant. (B) Proposed reaction mechanism for the cleavage of mRNA by RelE with residues from RelE shown in blue, 16S C1054 in green, and the mRNA in purple. Stacking of the second A site base with Y87 and the third base with 16S rRNA nucleotide C1054 (double arrows) first orients the RNA correctly for an inline attack. A high local concentration of positive charge shifts the pK_a_ of Y87 to allow it to act as a general base and abstract a proton from the 2′-OH promoting its attack on the phosphate between A site positions 2 and 3. The negatively charged bipyramidal transition state is stabilized by R61, while R81 acts as a general acid to protonate the 5′ OH leaving group, generating a 2′-3′ cyclic phosphate at the new 3′ end.

To clarify the involvement of the individual residues in catalysis, we constructed K52A, K54A, R61A, R81A, Y87A, and Y87F mutants of RelE and measured their cleavage efficiencies on *T. thermophilus* 70S ribosomes in vitro (Figure 5A). Based on our structures, we additionally designed a R81A/Y87F double mutant that we expected to have the strongest effect. Neither the lysine (K52A, K54A) nor the Y87F mutants showed a significant decrease in activity. However, mutation of either of the arginines (R61A, R81A) or the tyrosine (Y87A) significantly lowered the cleavage efficiency. Most remarkably, the R81A/Y87F double mutant almost completely abolished RelE activity. Together, these data indicate that both the arginines and the stacking of Y87 with the mRNA are important for the cleavage reaction and that the loss of the Y87 η-OH group can be compensated only when R81 is retained.

**Figure 6:**
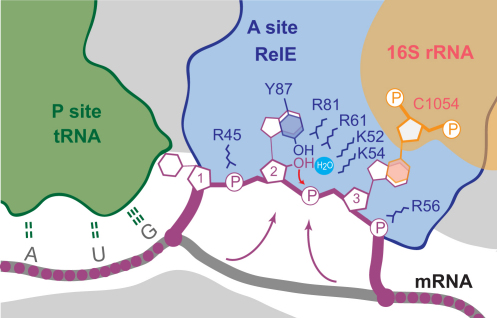
Overview of the RelE Cleavage Mechanism RelE (blue) occupies the A site where it pulls the mRNA (purple) into its active site (arrows). Here, the second nucleotide (“2”) stacks with Y87 (blue), while the third nucleotide (“3”) stacks with C1054 (orange). Relevant basic side chains and the water molecule in the active site are shown. The red arrow indicates the nucleophilic attack of the 2′-O^−^.

Both the structural and functional data thus indicate that Y87 and the arginines R61 and R81 are critically involved in catalysis. The lysines, K52 and K54, also have the potential to function in general acid/base catalysis, but our cleavage assay suggests that they are less important. However, they might contribute toward stabilization of the negatively charged transition state by analogy with RNase Sa2. The combined data therefore suggest a model for catalysis in which the mRNA is drawn into the active site by electrostatic forces from several basic side chains, while Y87 and 16S rRNA C1054 orient the substrate correctly for a nucleophilic attack of the 2′-OH of the second nucleotide in the A site on its own 3′-phosphate (Figure 5B). We propose that the high local concentration of positive charges in the active site lowers the pK_a_ of Y87 by stabilizing the negatively charged, deprotonated form. Y87 is then able to activate the 2′-OH by acting as a general base, possibly stabilized by hydrogen bonding to a water molecule observed at this location in the precleavage structure (Figures 4B, 6, and S1C). The reaction then proceeds toward inversion of the phosphate geometry via a negatively charged, trigonal bipyramidal transition state, most likely stabilized by R61 and possibly K54. A central role for R61 in transition state stabilization is consistent with the strong influence on RelE activity and the function of the corresponding R71 in RNase Sa2 (Bauerova-Hlinkova et al., 2009). Finally, R81 is in position to stabilize the leaving 5′-OH group by donating a proton during the final stage of the cleavage reaction and hence acting as a general acid (Figure 5B). One question that this mechanism raises is why the effect of the single mutations Y87F and R81A is so relatively weak when the combined effect of their mutation effectively abolishes the activity of the enzyme. It is possible that even in the absence of the Y87 OH group, the high concentration of arginine and lysine residues in the active site lowers the pK_a_ of the 2′-OH enough to alleviate the requirement for a specific general base. Furthermore, the side chain of Y87 is held in check by hydrogen binding to R81, so it is possible that lack of both the OH group and the R81 side chain leave the tyrosine in a conformation in which it is energetically unfavorable to stack with the critical second base of the codon. In summary, the structural and functional data presented here have allowed us to propose a detailed mechanism for RelE-mediated mRNA cleavage on the ribosome; however, a full understanding of all aspects of the reaction mechanism will probably require additional work by complementary techniques.

### The Sequence Specificity of the Reaction

RelE cleaves different codons with varying efficiencies, and although there appears to be no consensus sequence, most efficiently cleaved codons seem to follow the pattern Py-Pu-G, where Py is a pyrimidine and Pu a purine (Pedersen et al., 2003). In the precleavage structure, the first base of the A site codon is rotated back and contacts the P site tRNA (Figure 2E). This conformation would be difficult to achieve with a large purine base and could contribute to the subtle preference for pyrimidines at the first position. Likewise, the stacking interactions seen at positions 2 and 3 might be more stable in the presence of the larger purine bases. In the third position, the precleavage structure is consistent with an interaction between the guanine base and the region in RelE around E82, which could confer a more direct specificity toward G. However, none of these interactions seem essential for RelE binding, explaining why most codons are be cleaved with some efficiency, but they may explain some of the observed variations in activity.

### Conclusion

In this paper, we present crystal structures of the isolated ribosome-dependent endonuclease, RelE, and its complex with the ribosome in both pre- and postcleavage states. We present evidence that cleavage of mRNA on the ribosome is carried out by RelE and not by the ribosome itself, and we are able to propose a detailed reaction mechanism for RNA cleavage. These results have important implications for our understanding of the roles of RelE and other ribosome-dependent endonucleases in translational regulation.

The classical description of RelE as a toxin implies that it is a nonspecific inhibitor of translation, most compatible with a role in cell growth stasis or even cell death. However, the effect of RelE induction is more likely a reduction of the global rate of translation during amino acid starvation, leading to increased levels of charged tRNA and increased fidelity of translation (Christensen et al., 2001; Pedersen et al., 2002; Sorensen, 2001). This is supported by the observation that deletion of *relBE* and four other toxin-antitoxin loci encoding mRNA cleaving enzymes in *E. coli* significantly increases the global translational error rate, consistent with a role of RelE in quality control (K.G., unpublished data). The enzymes have more likely evolved to allow fast adaptation of bacterial cells to changing environments by modulation of the global rate of translation, and the term “toxin” is therefore misleading.

Ribosome-dependent endonucleases are widespread in free-living bacteria, and many are probably yet to be discovered. But sequence similarity can be very low between members of the family, so many are often only discovered after structural studies. One such example is RegB from bacteriophage T4, which was recently shown to be a member of the RelE superfamily by structural homology (Odaert et al., 2007). RegB participates in the life cycle of the phage by favoring the degradation of early mRNA with specific sequences inside the ribosome. It is therefore quite possible that the overwhelming number of RelE-like nucleases in bacteria act as “adaptation enzymes,” each appropriate for a specific environmental situation. Sequence-specific degradation of mRNA by ribosome-associated nucleases might therefore allow the control of translation as response to a range of conditions, including stress response, viral infections, and ribosome stalling.

## Experimental Procedures

### Preparation of RelE

A bicistronic construct encoding His-tagged RelB and untagged RelE-R81A mutant was synthesized (GenScript) and inserted into plasmid pMG25 (Christensen-Dalsgaard et al., 2008). The RelBE complex was expressed in *E. coli* BL21 DE3 (Novagen) and purified on Ni-NTA agarose (QIAGEN) equilibrated in 50 mM Tris (pH 8), 0.3 M NaCl, 5 mM MgCl_2_, and 5 mM 2-mercaptoethanol at 20°C. After wash with lysis buffer plus 15 mM imidazole, on-column denaturation in 50 mM Tris (pH 8), 0.3 M NaCl, 9 M urea, and 5 mM 2-mercaptoethanol allowed elution of free RelE-R81A. After shock refolding by 10-fold dilution into 50 mM Tris (pH 8), 10% glycerol, and 5 mM 2-mercaptoethanol, the protein was purified on a MonoS column (GE Healthcare) with a 0.05–1 M NaCl gradient followed by Superdex 75/300 GL gel filtration (GE Healthcare) in 15 mM Tris (pH 8), 0.1 M NaCl, and 5 mM BME, and concentration to 4–6 mg/ml on a spin filter (Milipore). The mutants used were generated by site-directed mutagenesis and purified similarly. For the ribosome complexes, the plasmid pSC2524HE was used for coexpression of His-tagged RelE and RelB (Christensen-Dalsgaard et al., 2008). The complex was denatured in 50 mM Tris (pH 8), 0.3 M NaCl, and 6 M guanidinium chloride while bound to Ni-NTA beads to remove RelB and renatured on column in 50 mM Tris (pH 8) plus 0.3 M NaCl. Subsequent purification steps were similar to the above. The RelE^R45A/R81A^ double mutant was constructed by site-directed mutagenesis.

### Structure Determination of Isolated RelE

Single crystals of untagged RelE grew up to 200 μm size by sitting drop vapor diffusion in 0.1 M Mes (pH 6.5), 0.2M (NH_4_)_2_SO_4_, and 30% w/v PEG 5000 monomethylether at 4°C. X-ray data to 2.5 Å were collected at the DESY X12 beamline (EMBL, Hamburg) from a single crystal flash-frozen in the mother liquor, and the data were processed with XDS (Kabsch, 1993). The structure was determined by molecular replacement in Phaser/PHENIX with a poly-alanine search model based on PDB 2KC9 (Li et al., 2009). A model covering residues 2–95 for three monomers was built in Coot (Emsley and Cowtan, 2004) and refined by iterative model building in PHENIX to a final R/R_free_ of 22.6% / 26.8%.(Adams et al., 2002).

### Preparation of 70S Ribosomes, tRNA, and mRNAs

*T. thermophilus* ribosomes and *E. coli* tRNA^fMet^ were purified as described previously (Selmer et al., 2006). mRNAs with the sequence 5′-GGCAAGGAGGUAAAAAUGUAGAAAA-3′ were synthesized with appropriate modifications, phosphorothioate linkages after nt 19 and 20 (MAP), 2′-O-methylation at nt 19 (MAO1), 19 + 20 (MAO2), or 19 + 20 + 21 (MAO3), or a 2′ deoxy ribose at nt 19 (MAD), while “Trunc Asite” had the sequence 5′-GGCAAGGAGGUAAAAAUG-3′.

### Structure Determination of Ribosome-RelE Complexes

Complexes of RelE^wt^ or RelE^R45A/R81A^ with the 70S ribosome, tRNA^fMet^, and unmodified or MAO3 mRNA were crystallized as described previously (Selmer et al., 2006). All complexes were formed in 5 mM HEPES (pH 7.5), 10 mM Mg(CH_3_COO)_2_, 50 mM KCl, 10 mM NH_4_Cl, and 6 mM 2-mercaptoethanol. In brief, 70S ribosomes at a final concentration of 4.4 μM were incubated with a 2-fold excess of mRNA and a 2.5-fold excess of tRNA^fMet^ at 55°C for 30 min. RelE was added in 5-fold excess and incubated for 10 min at 37°C and 30 min at 20°C. Crystals were grown by sitting-drop vapor diffusion as 2.4 μl complex including 2.8 mM Deoxy Big Chap (Calbiochem) plus 2 μl reservoir solution containing 0.1 M Tris-HAc (pH 7.2), 0.2 M KSCN, 3%–4.5% w/v PEG 20K, and 3%–4.5% w/v PEG 550 monomethylether and left to equilibrate at 20°C. The crystals grew within 2 weeks to a size of up to 80 × 100 × 600 μm and were gradually transferred into cryoprotecting solution (0.1 M Tris-HAc [pH 7.2], 0.2 M KSCN, 10 mM NH_4_Cl, 10 mM MgAc, 5% PEG 20K, and 25% PEG550MME), and frozen in liquid nitrogen for data collection at 100 K.

### Data Collection, Refinement, and Model Building

Crystals were screened at beamlines ID14-4 at the European Synchrotron Radiation Facility (ESRF) in Grenoble, France, and X06SA PXI at the Swiss Light Source (SLS), and data were collected from suitable crystals at six positions on four crystals for the precatalytic state and at seven positions of two crystals for the postcleavage state at PXI (SLS) and processed with XDS (Kabsch, 1993). An empty 70S model was used for initial refinement and phase calculation (Voorhees et al., 2009) using both CNS (Brunger et al., 1998) and Phenix (Adams et al., 2002) as described previously (Selmer et al., 2006; Weixlbaumer et al., 2008). 3mF_o_-2DF_c_ and mF_o_-DF_c_ difference maps were used for model building of initially P site tRNA^fMet^, noncognate E site tRNA^fMet^, and the mRNA except the A site codon. The ribosome model was modified by comparison with a recent, more complete model (Gao et al., 2009). Changes in the 16S rRNA at the decoding center and near C1054 were manually fitted, and, finally, the models of mRNA and RelE were built starting with the isolated RelE crystal structure. The final refinement in CNS had R/R_free_ of 21.9% / 24.7% and 21.5% / 24.8% for the precleavage and postcleavage structures, respectively.

### Analysis of Cleaved mRNAs

All mRNAs were 5′ end labeled with T4 polynucleotide kinase and ATP-γ^32^P and complexed formed essentially as described above but only with 3.5 μM mRNA. Reactions contained a 5-fold excess of RelE and were incubated for 10 min at 37°C followed by 30 min at 20°C. For the mutagenesis screen, 2.2 μM of each RelE mutant (0.5-fold) was incubated in triplicate independent reactions with 4.4 μM programmed 70S ribosomes formed in polymix buffer (Jelenc and Kurland, 1979) for 15 min at 37°C. The reactions were stopped by addition of phenol/chloroform, the RNA extracted and concentrated with ethanol precipitation, and separated by 18% urea denaturing PAGE. Bands were recorded on a phosphor imaging plate, visualized with a Typhoon scanner, and quantified with Image Quant TL (both GE Healthcare). For mass spectrometry, samples were prepared with unlabeled mRNA and incubated with and without RelE^wt^. After gel purification and precipitation, the dried pellets were resuspended in water, desalted, and prepared with ZipTip C18 pipette tips (Millipore) according to the instructions of the manufacturer and analyzed with an Ultraflex III MALDI TOF/TOF spectrometer (Bruker Daltonics) in positive ion, linear mode (Spottke et al., 2004).

## Figures and Tables

**Table 1:** Summary of Crystallographic Data and Refinement

	70S-RelE Complex Precleavage State	70S-RelE Complex Postcleavage State	Isolated RelE
Data Collection Statistics			
Space group	P2_1_2_1_2_1_	P2_1_2_1_2_1_	P12_1_1
Unit cell dimensions			
a, b, c, Å	a = 211.6, b = 452.0, c = 622.4	a = 211.2, b = 451.4, c = 623.3	a = 40.35, b = 60.79, c = 70.32
α, β, γ	α = β = γ = 90	α = β = γ = 90	α = γ = 90, β = 104.53
Resolution, Å	50–3.3 (3.4–3.3)	50–3.6 (3.7–3.6)	19.5–2.5 (3.0–2.5)
R_sym_, %	16.3 (119.3)	22.9 (114.1)	9.6 (47.4)
I / σ_I_	9.80 (1.95)	8.39 (2.11)	11.8 (3.6)
Completeness, %	99.8 (99.6)	99.8 (99.7)	89.4 (81.7)
Redundancy	8.46 (7.43)	8.47 (7.26)	3.0 (2.4)

Refinement Statistics			

Resolution, Å	50.0–3.3	50.0–3.6	19.5–2.5
Number of unique reflections	883,810	681,993	11,259
R_work_ / R_free_, %	21.9 / 24.7	21.5 / 24.8	22.6 / 26.8
Molecules per a.s.u.	2	2	3
Number of atoms			
RNA	99,764 (per molecule)	99,751 (per molecule)	−
Protein	47,875 (per molecule)	47,889 (per molecule)	2,333
Ions	536 (per molecule)	536 (per molecule)	−
Solvent	−	−	18
Average B factor			
RNA	113	106	−
Protein	123	117	67.9
Rmsd from ideality			
Bond lengths (Å)	0.007	0.007	0.009
Bond angles (°)	1.17	1.19	1.36

X-ray data and refinement statistics. Numbers in parentheses indicate the values for the outermost resolution shell.

## Accession Numbers

Coordinates and structure factors have been deposited in the RCSB Protein Data Bank with accession IDs 3KHA (isolated RelE); 3KIQ, 3KIR, 3KIS, and 3KIT (70S-bound RelE, precleavage state); and 3KIU, 3KIW, 3KIX, and 3KIY (70S-bound RelE, postcleavage state).

## Supplemental Data

Document S1. Four Figures
