# Supplementary material for: The Structural Basis for mRNA Recognition and Cleavage by the Ribosome-Dependent Endonuclease RelE
Source: Cell. 2009 Dec 11;139(6):1084–95. doi: 10.1016/j.cell.2009.11.015 (PMC2807027; doi:10.1016/j.cell.2009.11.015)
Supplement: Document S1. Four Figures [file mmc1.pdf]

Cell, Volume *139*

Supplemental Data

The Structural Basis for mRNA Recognition and Cleavage by the Ribosome-Dependent Endonuclease RelE

Cajetan Neubauer, Yong-Gui Gao, Kasper R. Andersen, Christine M. Dunham, Ann C. Kelley, Jendrik Hentschel, Kenn Gerdes, V. Ramakrishnan, and Ditlev E. Brodersen

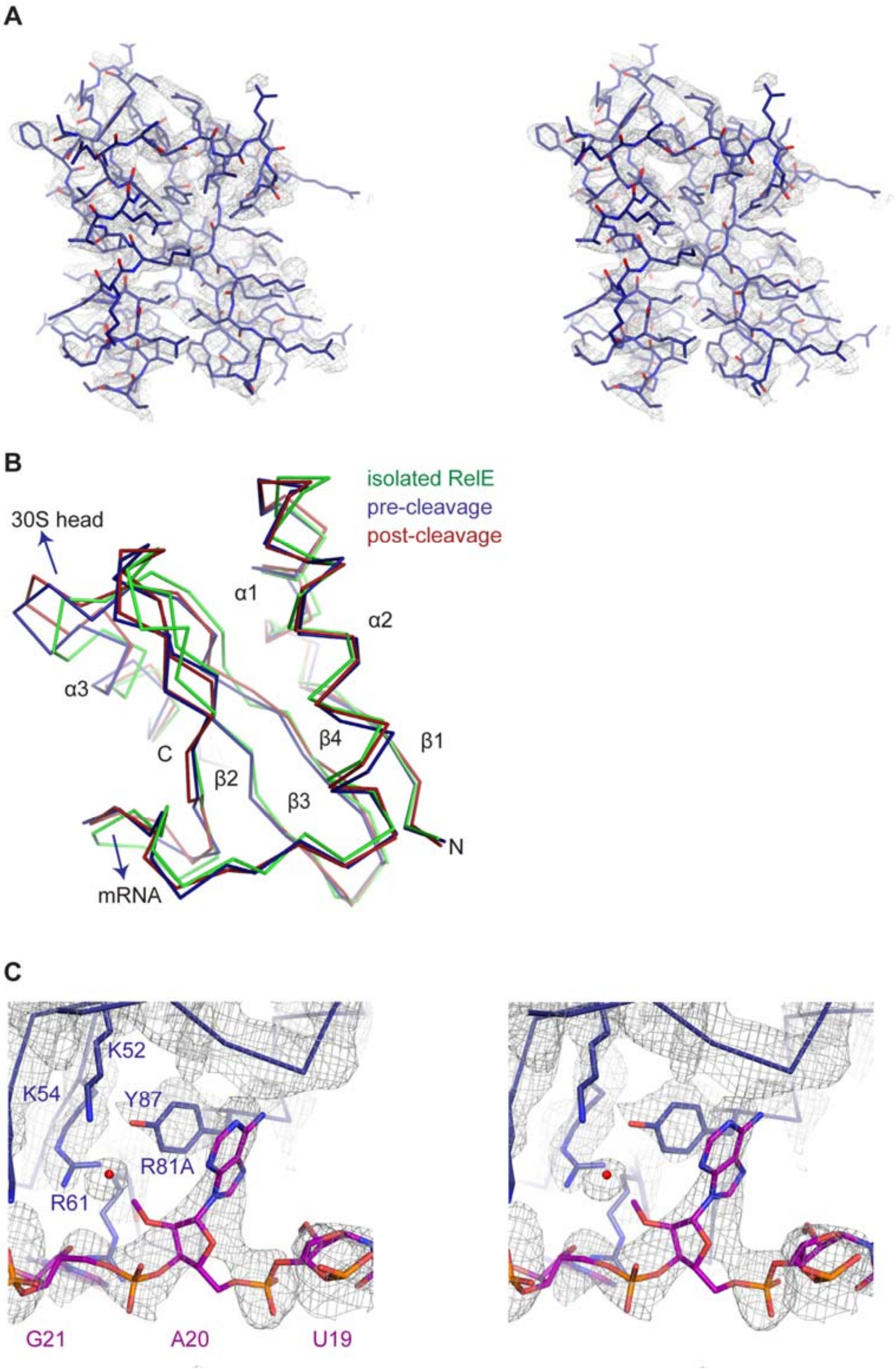

Figure S1. Electron density and structural comparison. A. Unbiased $3DF_o$-$2mF_c$ RelE omit map (contoured at 1.5σ) showing electron density for RelE inside the ribosome in the pre-cleavage structure. **B.** Structural comparison between the crystal structures of isolated RelE (molecule A, green), and the ribosome-bound forms in the pre-cleavage (blue) and post-cleavage (red) states. N and C termini, secondary structure elements, and regions of interaction are shown. **C.** Stereo view of the final, refined mF_o-DF_c electron density map around the mRNA region in the pre-cleavage state, contoured at 1.2σ.

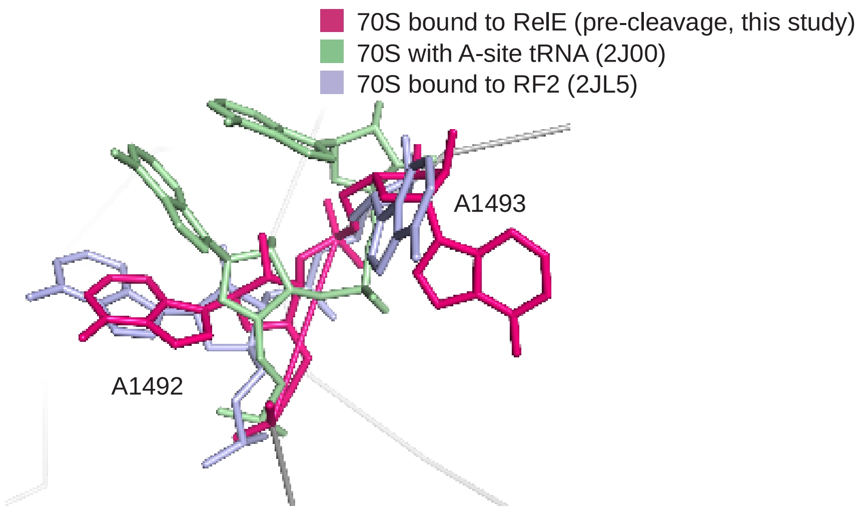

Figure S2. Conformation of the decoding site. Conformation of the decoding centre adenosines A1492 and A1493 in a number of ribosome complexes including the 70S bound to RelE (purple, this study), the 70S bound to A-site tRNA (PDB 2J00 (Selmer et al., 2006)), and the 70S bound to release factor 2 (PDB 2JL5 (Weixlbaumer et al., 2008)).

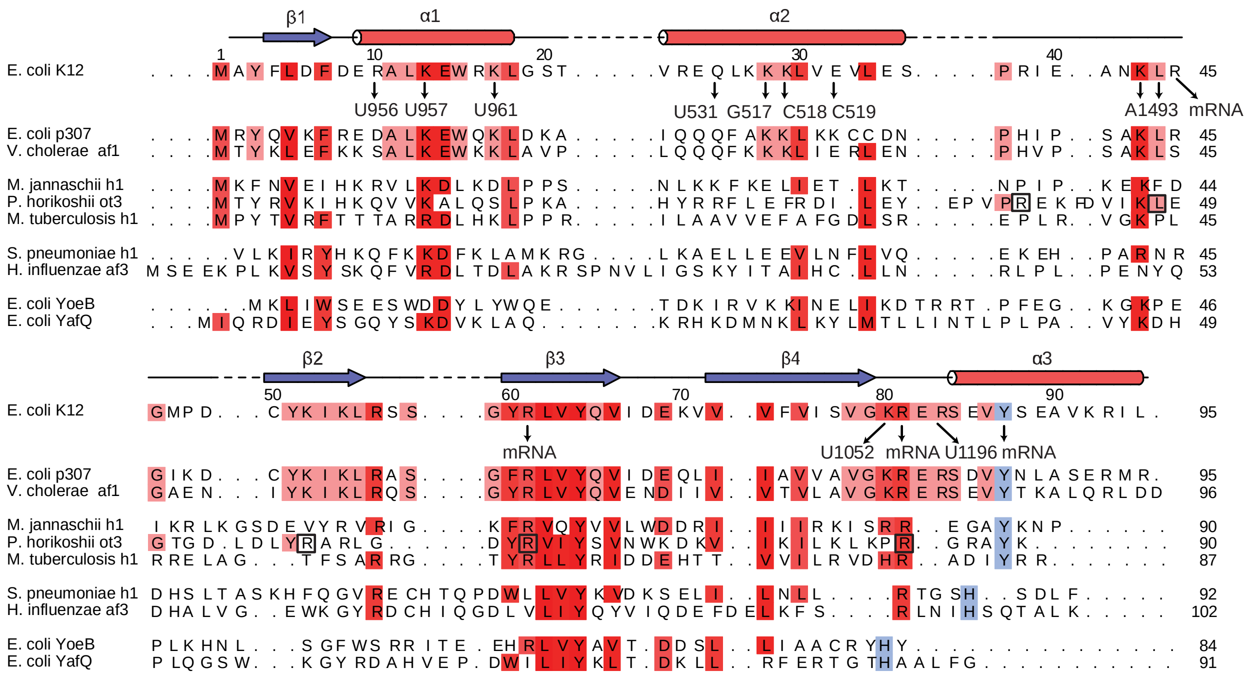

Figure S3. Sequence alignment of RelE and homologues. Sequence alignment of the genomic *E. coli* K12 RelE (used for this study) with RelE homologues from a range of archaea and pathogens. *E. coli* p307, a plasmid-borne homologue, *Vibrio cholerae* af1, *Methanococcus jannaschii* h1, *Pyrococcus horikoshii* ot3 with known structure (Takagi et al., 2005), *Mycobacterium tuberculosis* h1, *Streptococcus pneumoniae* h1, *Haemophilus influenzae* af3, and the related toxins, YoeB with known structure (Kamada and Hanaoka, 2005) and YafQ from *E. coli*. The alignment is based on sequence, and for *P. horikoshii* ot3, the available structure (Takagi et al., 2005). Highly conserved residues are shown with increasing strength of red color, and the conserved tyrosine or histidine at the C-terminus with light blue. The secondary structure of *E. coli* RelE is shown above the sequences and contacts to the rRNA or mRNA below the RelE sequence (all rRNA numbers correspond to the *E. coli* 16S). Residues in the *P. horikoshii* RelE homologue that significantly reduces the inhibitory activity when mutated to alanine are indicated with black boxes.

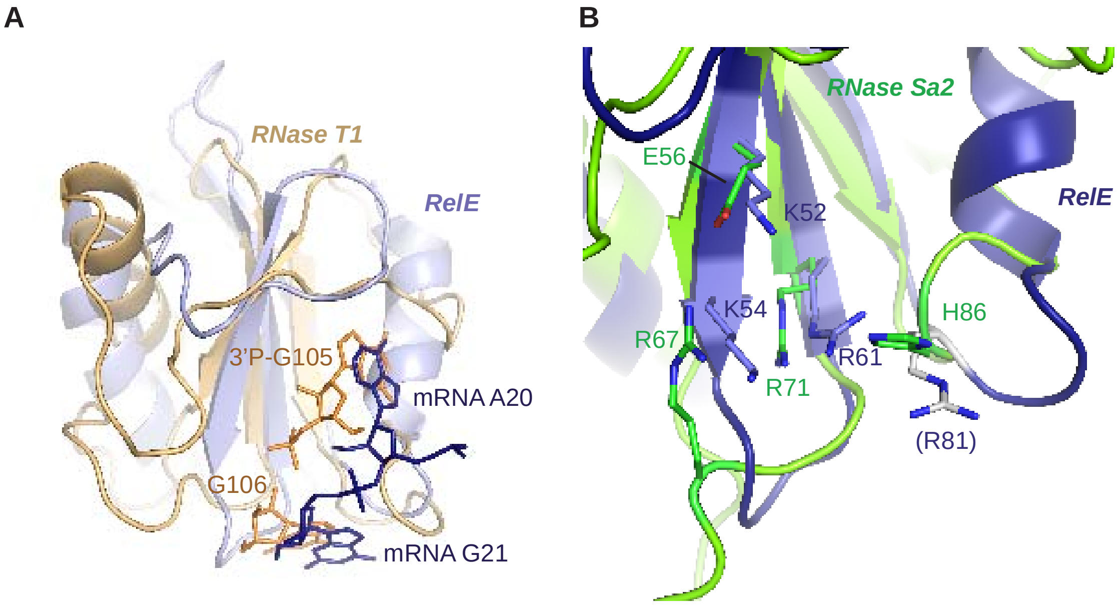

Figure S4. Comparison of RelE with RNase T1 and RNase Sa2. A. The structure of RNase T1 (light orange, PDB 1RGA (Zegers et al., 1994)) superimposed on the ribosome-bound structure of RelE in the pre-cleavage state (light blue) with the two guanosine residues in the RNase T1 structure and A-site codon positions 2 (A20) and 3 (G21) of the present structure shown as orange and blue sticks, respectively. **B.** Structural superposition of RNase Sa2 (green, PDB 3D5I (Bauerova-Hlinkova et al., 2009)) and RelE in the pre-cleavage state (blue) showing equivalent positions of important residues.
